# GH treatment in pediatric Down syndrome: a systematic review and mini meta-analysis

**DOI:** 10.3389/fendo.2023.1135768

**Published:** 2023-04-21

**Authors:** David Shaki, Eli Hershkovitz, Shai Tamam, Arkadi Bollotin, Odeya David, Guy Yalovitsky, Neta Loewenthal, Lior Carmon, Dganit Walker, Raphael Nowak, Alon Haim

**Affiliations:** ^1^ Pediatric Endocrinology Unit, Soroka University Medical Center, Beer Sheva, Israel; ^2^ Faculty of Health Sciences, Ben-Gurion University, Beer Sheva, Israel; ^3^ Library of Life Sciences and Medicine, Tel Aviv University, Tel Aviv, Israel

**Keywords:** growth hormone, GH, rGH treatment, Down syndrome, insulin-like growth factor 1, IGF1

## Abstract

**Objective:**

To analyze and determine the safety and efficacy of growth hormone (GH) treatment in Down syndrome (DS) pediatric patients and to weigh ethical aspects involved.

**Design:**

Systematic review and mini meta-analysis of the literature.

**Methods:**

A search was performed in PubMed, Embase, Scopus, and PsycINFO through August 2022. Eligible studies included those who answered at least one of the following two questions: 1) What is the effect of growth hormone treatment in children with Down syndrome? 2) What are the ethical arguments in favor and against growth hormone treatment for children with Down syndrome? Multiple reviewers independently screened each article for eligibility.

**Results:**

In total sixteen reports detailed medical effects of GH treatment in pediatric DS patients and eight studies dealt with ethical aspects of GH treatment. Treatment with GH resulted in significantly higher growth velocity in patients with DS. The ethical complexity is great but does not present insurmountable difficulties to the therapeutic option.

**Conclusions:**

As GH treatment is safe and effective for short-term height growth, GH therapy should be considered in long-term treatment of DS children.

## 1 Introduction

Down syndrome (DS) is the most common chromosomal disorder with an incidence of one in 700 live births in the United States (1) and 1–10 in 1,000 live births worldwide, according to the WHO (2). Linear growth retardation is a cardinal characteristic of DS. Pathologic low height velocity is mainly marked in infancy and adolescence (3). A significant portion of short-stature pathogenesis in children with DS is associated with impaired GHRH-GH-IGF1 axis function, mainly due to an abnormal quantitative and qualitative capacity of the hypothalamus–pituitary axis and reduced bioactivity of endogenous growth hormone (GH) (4).

The effect of GH therapy in children with DS on height, head circumference, and cognitive and motor function has been tested in research studies since the 1950s. Various and even conflicting findings have been reported, and in the absence of a systematic review, it is difficult to establish well-founded conclusions on its true, overall effect.

Life expectancy for people with DS continues to rise – the median lifespan is now 58 years with many living into their sixties and seventies (5). Heart conditions, which can accompany DS, have been routinely corrected by surgery since the early 1980s. Consequently, longer life expectancy also raises the importance for DS patients to have a good quality of life.

This study reviews for the first time all the research reported data with references to the effect of GH therapy for children with DS on longitudinal growth, head circumference, cognitive and motor function, insulin-like growth factor 1 (IGF1) levels, bone age, as well as short and long-term treatment side effects. In addition, we review all data with references to the ethical aspects of GH therapy in DS patients.

## 2 Methods

### 2.1 Search strategy and study selection

The present systematic review was performed in accordance with the Preferred Reporting Items for Systematic Reviews and Meta-Analyses (PRISMA) guidelines (6).

No formal ethical approval was required. An extensive literature search of four electronic databases: PubMed, Cochrane Library, Scopus, and PsycINFO *via* EBSCO was undertaken for studies regarding DS and growth hormone, published until January 2021. The general keywords were ‘Down syndrome’ and ‘growth hormone’, while the search strategy was updated and adapted for each database. Vocabulary supplemented with keywords were used for searches that were conducted separately for each topic and combined later. The search was restricted to humans, and no other restriction was made. Studies in all languages were included. Full-text articles of potentially relevant studies not available through the university library were requested from the authors. We repeated the search on September 2022 and received 27 additional records. A review of the title or abstract was enough to determine that they are not suitable for inclusion in this review.

### 2.2 Quality assessment and risk of bias

The scope of data reporting in much of the original works did not allow for a full quality and risk of bias assessment to be carried out on the individual original studies; therefore, no individual quality assessment was carried out.

We used ROBIS, a tool for assessing quality and risk of bias in systematic reviews. The tool is completed in three phases: assess relevance (optional), identify concerns with the review process by 21 questions divided to 4 domains, and judge risk of bias in the review. It is the first rigorously developed tool designed specifically to assess the risk of bias in systematic reviews (7).

### 2.3 Eligibility criteria

Eligible studies enrolled pediatric DS patients who were treated with GH during childhood and examined the effect of treatment on longitudinal growth and other aspects or dealt with the ethical aspects of such treatment. Studies that answered one of the following two questions were included:

1) What is the effect of growth hormone treatment in children with Down syndrome? In relation to this question, studies that examined the effect of such treatment on at least one of the following six outcomes: height, head circumference, cognition and motor skills, side effects, bone age, and IGF1 level, were included.
2) What are the ethical arguments in favor and against growth hormone treatment for children with Down syndrome? In relation to this question, studies that made claims in at least one of the following three categories: safety of GH treatment, necessity for GH treatment, and agreement and autonomy, were included. Two reviewers (D.S. and G.Y.) worked in duplicate independently and extracted data on study characteristics and outcomes. Studies were eligible for inclusion regardless of design, language, year, or sample size.

### 2.4 Data extraction

Data extraction included a full description of participants if available. Foreign language articles were translated by multilingual reviewers. The main outcome extracted from studies for mini meta-analysis were GH treatment characteristics, during and post-treatment height effects, head circumference, cognitive and motor function, additional therapeutic effects, adverse effects, IGF1, bone age response, and ethical arguments in favor and against GH therapy in DS patients. Additionally, we collected the number and age of the participants in the research groups as well as in the control groups, inclusion criteria, type of dose, and duration of growth hormone treatment.

### 2.5 Statistical analysis

We used a random effects meta-analysis in order to assess heterogeneity. We assessed the degree of inconsistency in the results between studies using the I² statistic; this statistic explains the proportion of inconsistency between studies that cannot be explained by chance alone, and is likely due to real differences in the population or the conduct of the studies (8).

Height standard deviation scores (SDS) were estimated in each study and pooled using a random effects meta-analysis (9). SDS expressed height differences in terms of the SD for the height of the reference population. Most studies reported SDS in reference to their national databases. To compare the change in height SDS along time between GH treated and untreated DS patients and also between DS patients with and without proven growth hormone deficiency under GH treatment, we extrapolated data using studies in which we could extract mean and standard deviations for all children in the different time points. We used two-way repeated-measures ANOVA to assess the difference between groups over time. R statistical programming language was used for extrapolation and statistical analysis. Publication selection bias could not be calculated due to missing relevant data.

## 3 Results

### 3.1 Search results

**Figure 1:**
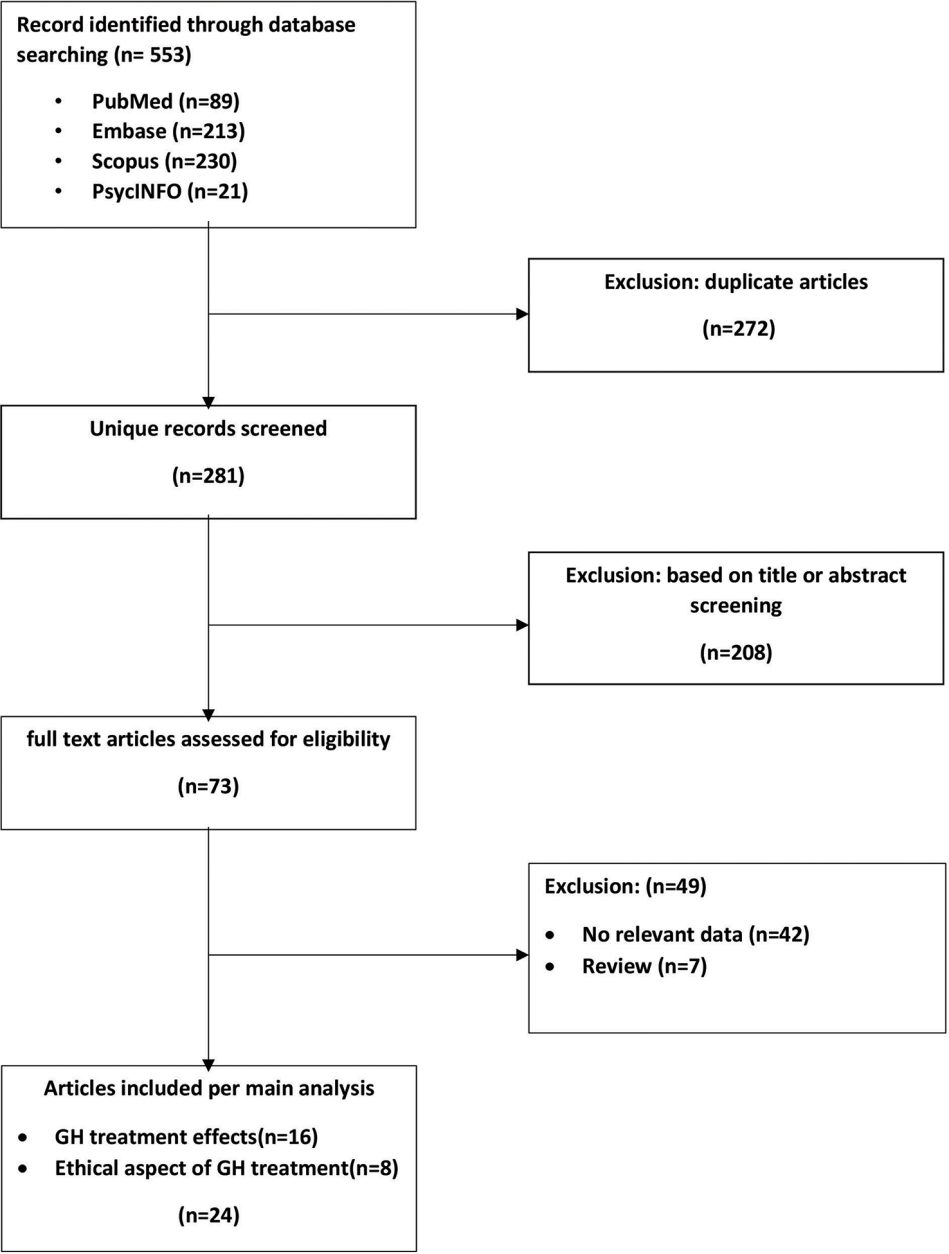
Flowchart of article screening and inclusion.

An initial search of the literature yielded 281 publications and 24 eligible studies (**Figure 1**). Sixteen reports detailed medical effects of treatment with GH. Some are follow-up studies reporting late treatment outcomes or completing statistical analyses that did not appear in the initial report. Eight studies dealt with the ethical aspects of GH treatment in DS patients. The main characteristics and findings of the included studies are presented in **Supplementary Table 1** and **Table 1**.

**Table 1:** Arguments regarding the ethical aspect of growth hormone treatment in children with Down syndrome.

Study	Arguments		
	Safety of GH treatment	Necessity for GH treatment	Agreement, justice, and autonomy
Binder ND., 1992 (10)	Previous study of GH treatment was completed in a small group without any significant short-term side effects.	Missing a clear statement on the benefits of treatment for DS patients with GH. Raised questions on: what were the benefits to being taller for these patients?, and do benefits outweigh inconvenience or discomfort of the treatment?	Questions whether the researchers believe that it is obligatory to treat DS children with GH? Is the treatment required even if it is against the wishes of the parents?
Underwood LE., 1992 (11)	Authors fail to express concern about leukemia in GH-treated children with DS. Especially important as DS patients have a predisposition to leukemia.	Recommendation for studies to assess child’s biologic and psychologic need for GH, the potential benefits to the child, and the potential physical and psychological risks of treatment. Should question belief that “taller is more desirable”. Implied that head circumference provides special benefit to patient but did not show that increased head size related to improved intellectual performance. Parents might be misled and pursue therapy for the wrong reasons.	Physicians and parents should be wary before controlled studies are completed. Informed consent is difficult because many of these children are limited in their ability to understand issues involved with GH treatment.
Allen D., 1992 (12)	Analysis of potential risks of treatment should include development of leukemia in a population already at increased risk.	Goal of GH therapy is not tall stature, but rather improved quality of life resulting from having achieved height within the normal range. Evidence needed that short stature is a disability for a particular individual. Concern of psychologic effects of repeated injections, especially for children not understanding purpose of treatment. Disability related to height and likelihood of therapeutic benefit should guide therapy.	Assertion that GH responsiveness justifies treatment with GH is oversimplified, and specific diagnosis should not be an automatic indication for GH therapy.
Lantos JD., 2000 (13)		Relationship between GH and growth in DS patients has been established, but is height increase only of value because of perception of others? DS children may not experience enhanced sense of well-being by attempting to fit an externally defined ideal. The more GH treatment can be shown to produce benefits other than height, the more justifiable its use will be.	
Allen DB, Frasier SD, Foley TP, Pescovitz OH., 1993 (14)	Although recombinant human GH appears to be safe, analysis of the risks of GH therapy in children with DS must include the possible development of leukemia, and risks not foreseen at present.	Neither short-term nor long-term therapeutic trials of GH therapy have demonstrated improvement in psychologic, intellectual, or social development of children with DS. There is no evidence that an increase in head circumference with GH treatment can be equated with improved intellectual performance by children with DS. Prospective placebo-controlled studies are needed to determine efficacy of GH in improving growth and functional capacity. Evidence that short stature is a disability for a particular individual should also be required. Does short stature per se impair increasingly improved outlook for socialization and employment?	Therapy should be based on child’s disability in relation to height and benefit, and not simply on diagnosis.
Castells S, Wisniewski KE., 1994 (15)	Each child receiving GH is routinely assessed with complete blood cell count and chemistry profile and monitored for: hypothyroidism, slipped capital femoral epiphysis, hyperglycemia, leukemia, and pseudotumor cerebri.	Researchers recognize that all children receiving GH should be in controlled studies to obtain information on effects on growth, head size, facial characteristics, and functional capacity, with special attention to intellectual or social development.	In all cases, parents are well informed of the known risks and benefits of GH treatment, diagnostic procedures are discussed, and a consent form is signed.
Duffey DL., 1994 (16)	Research in England reported that cancer patients receiving human GH treatment experienced 50% less recurrence of tumours than the children who were not receiving GH (Ogilvy-Stuart, 1992)	As inclusion in the normal classroom and realisation of potential growth for children with DS, benefits of normal height and growth and appearance become just as important for children with DS as for any other child. If ignored, risk layering handicaps on top of handicaps and depriving children with DS any chance of living a healthy and happy life as contributing members of society.	Treatment from child should not be withheld simply on basis of presence of disability (Child Abuse Amendments of 1984). It is ethically, morally, and legally right that every child with DS or disability receives same treatment as any other child, without bias or judgment.
Kodish E, Cuttler L., 1996 (17)	Risk of acute leukemia is elevated in all children with DS, and for this, the leukemogenic potential of GH therapy should be considered when balancing the risks and benefits.	There are data to suggest that psychosocial morbidity is associated with short stature in other groups of children but cannot be directly extrapolated to DS. GH may make children with DS stronger and taller, but these outcomes are not likely to decrease their morbidity or mortality. Children with DS are affected by many factors that contribute to morbidity (multifactorial etiologic contributors). The extent to which a child may perceive psychologic or functional morbidity and feel and appreciate benefit of treatment must be considered separately for each case. Measures are needed that assess functional benefit or functional outcomes of treatment; quantitative measures lack practical relevance, unless accompanied by an understanding of their functional impact. Major arguments for: accelerated growth velocity, increased height, and possibly improved muscle strength.	Pediatricians needs to respect and consider experiences of parents and their wishes regarding decisions about GH therapy. This does not relieve physicians of their obligation to the children under their care. Pediatricians should carefully discuss potential risks and benefits of GH therapy with parents of children with DS.
“Little bigger, little better” Lancet Article, 1994 (18)	Do not know about possible side-effects, for example: leukemia, effects on the CNS and skeleton, glucose tolerance, pubertal maturation, and final adult height.	Arguments in favor of therapy include a perceived need for extra height to help DS children feel “normal” in an uncaring world and a suggestion of improvement in general wellbeing. Trials show that at 2 years all 16 enrolled patients with GH therapy were above the 95 percentile for DS children. At 3 years there was no drop in growth velocity.	

### 3.2 Mini meta-analysis-GH treatment

#### 3.2.1 Height outcome

Most studies present height data by using average height SDS and growth curves of a normal population, while some also present the average growth velocity. The problem with using growth velocity is the normal variation in this parameter is dependent on the age, gender, and pubertal status of the patient.

One important study presented height data by using a percentile range for each patient at the start and end points of a GH treatment period (19). Another study presented height data by using growth velocity alone (20). Unfortunately, it was impossible to utilize both studies’ essential height data within the mini meta-analysis. Nonetheless, both studies presented an impressive longitudinal growth response to GH therapy over three years of treatment in children with DS. Only one study showed continuation of GH treatment in a small proportion of children until they reached their final height.

A control group was recruited in two studies (21, 22) However, detailed control group height data were not presented and, therefore, one was not included in the statistical analysis (21). No GH deficiency diagnosis was required in three of the four studies included in the mini meta-analysis (21–23). A diagnosis of GH deficiency was required in one study included in the meta-analysis (24), as well as in two other studies that could not be included (19, 20).

**Figure 2:**
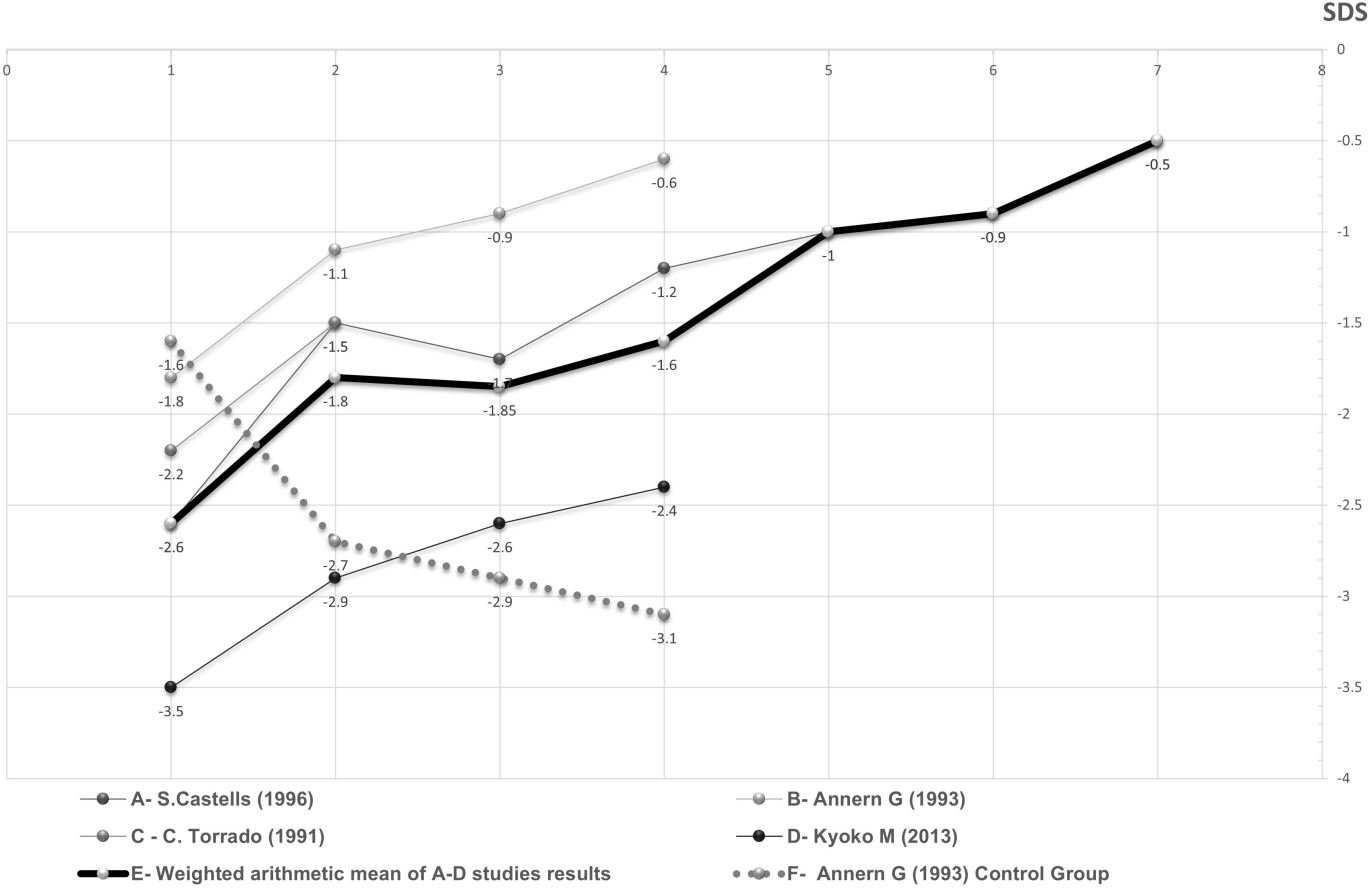
Periodic average height SDS of DS patients during GH treatment as a function of duration of treatment in years. The fragmented and continuous lines represent different DS patients groups whose source data appear in the studies mentioned in the figure itself. The thickened line represents the weighted arithmetic mean of GH treated groups. The dotted line represents the control group.

Significant difference in the change in height SDS over time was found between GH treated and untreated DS patients. (Means of -1.22 and 0.81, p-value<0.0001) (**Figure 2**). No statistically significant difference in the change in height SDS over time was found between children with and without proven GH deficiency (means and standard deviations of 0.86 +/- 1.2 and 0.78 +/- 1.4, p-value=0.73)

The description of height response to GH therapy in all studies is described in **Supplementary Table 1**. Height SDS data of DS children treated with GH in the four studies providing this parameter, as well as their weighted average and control group data, are presented in **Figure 2**. 

Risk of bias of systematic review was evaluated according to ROBIS. Phase 2 includes 3 essential domains: identification and selection of studies, data collection and study appraisal, and synthesis and findings. 14 signaling questions in three domains were corresponded to “low risk of bias” while 2 signaling questions, one in domain 3 and one in domain 4, were classified as “no information,” and hence corresponded to “unclear risk of bias”.

**Figure 3:**
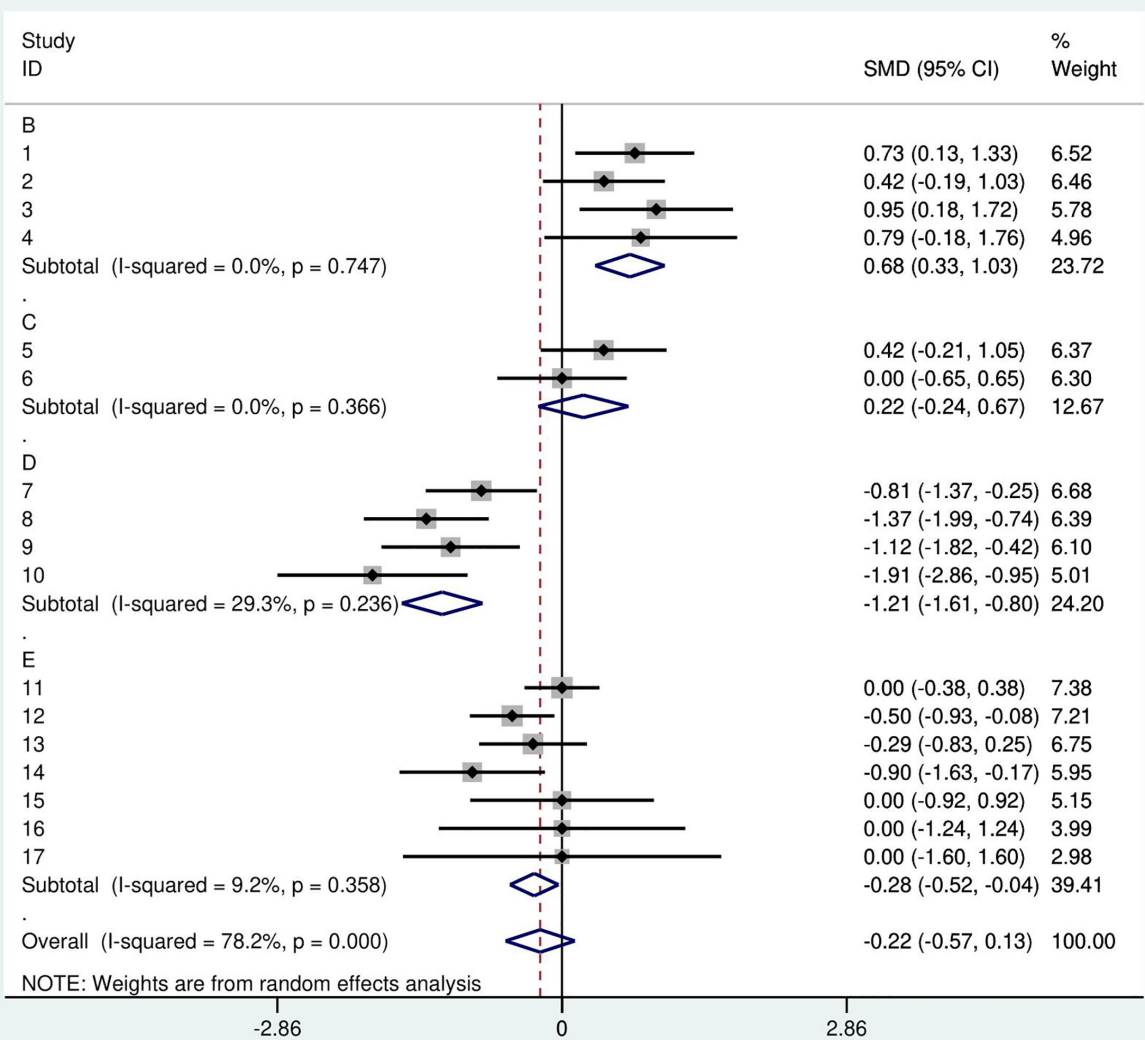
A forest plot of the GH therapy mini meta-analysis by study. Random effects meta-analysis of height SDS was performed in comparison to the reference group. Squares- point represent the result of each study, each year of GH treatment, or follow-up. A- Castells S. 1996. B- Annerén G.1993. C- Torrado C. 1991. D- Meguri K 2013. E- weighted arithmetic mean of GH treated groups, F- Annerén G. 1993 control group.

Heterogeneity index I-squared was calculated to be 78.2%. The estimate of between-study variance tau-squared was 0.3897. A forest plot of the mini meta-analysis is presented in **Figure 3**.

#### 3.2.2 post-treatment effect on height

One study reported that growth velocity returned to baseline after stopping treatment (25). In one study, the predicted adult height (PAH) was calculated during therapy, and was found to be 170.7 ± 10.8 cm in 19 males and 167.1 ± 5.2 cm in 16 females. PAH was normalized in 91% of the children receiving treatment (21). A series of consecutive studies showed that the accelerated growth benefit was maintained for the short term, three years from the end of treatment (26), but was not maintained for the long term, ten years from the end of the treatment. Final height in those patients did not differ between the GH-treated subjects and the extended group of controls (27). Another study showed that the benefit of growth is maintained even 10–15 years from the end of treatment, with an improvement in the final height on average of 5.16 cm in males and 7.35 cm in females, compared to C. Cronk DS growth charts (20).

#### 3.2.3 Head circumference

All studies investigating the effect of recombinant human GH treatment in the early years of life on head circumference showed a positive effect (21–23, 27, 28). The degree of impact reported ranges from very slight improvement to dramatic improvement and normalization of the head circumference.

### 3.3 Cognitive function

There was a significant change in cognitive function compared to the control group in one study alone. Approximately 15 years after GH therapy termination, there was no statistical preference to the GH therapy group in brief IQ scores, but significantly higher scores in all subtests of cognitive tests Leiter-R and WISC-III and in all but one subtest of the motor BOT-2 test (27). The other studies that tested cognitive function found no change (25, 29–31).

#### 3.3.1 Motor function

One study showed an improvement in fine motor performance in the short term (30) and in all but one subtest of the motor BOT-2 test in the long term (27). One case report showed a dramatic improvement in both fine and gross motor function (32).

### 3.4 Additional effects

One study showed possible craniofacial growth and dental maturation that may be associated to the treatment (31).

Although the length of menstrual cycles in DS is within the normal range, a reduction in reproductive function is generally observed. A study examining the function of HPG axis showed reduced sensitivity of ovaries to FSH in patients with DS compared to controls. A treatment with GH normalized the ovarian response to FSH (33).

#### 3.4.1 IGF1 levels

Almost all studies examined the response of IGF1 level to growth hormone treatment and showed that IGF1 levels in plasma were below normal range before treatment and increased to normal range shortly after starting treatment and remained normal throughout treatment.

#### 3.4.2 Bone age

Bone age was delayed before treatment began. Some studies showed advances in bone age during treatment (21, 25, 26, 29), and others showed no significant change (22–24).

### 3.5 Adverse effects

Two studies reported an increase in mean neutrophil lobe count (25, 34). One study also showed an increase in neutrophil count during treatment and a fall thereafter (25). One study (three articles) reported on a single child with slightly elevated liver enzymes (22, 25, 29). Another study reported on mild subclinical hypothyroidism (19). Precocious puberty was observed in one boy 3.4 yrs after GH treatment initiation (24). In no study was the development of leukemia reported.

### 3.6 Ethical considerations of GH therapy in DS

The ethical legitimacy of GH therapy in children with DS has been widely questioned over the years. The claim put forth in Torrado et al. (23), which argues that responsiveness to GH therapy by DS children justifies prescribing a course of treatment, was challenged by several respondents.

Various arguments have been presented for and against the treatment. These arguments can be divided into 3 categories: safety of GH treatment, necessity for GH treatment, and agreement, justice, and autonomy. A summary of all arguments that have appeared in the various studies on this subject appear in **Table 1**.

## 4 Discussion

### 4.1 GH treatment

Consistent with the results of this meta-analysis, it can be concluded that there is a significant short-term beneficial effect of GH therapy on longitudinal growth in children with DS. The growth velocity of patients with DS treated with GH was found to be significantly higher than in non-treated DS patients.

Except for a single study that reported on the maintenance of the therapeutic effect even after stopping treatment, all the other studies show that the effect of short-term treatment gradually fades after the end of treatment. Importantly, there is almost no data on the effect of long-term treatment. It is possible that long-term treatment until the end of growth may preserve and even increase the therapeutic effect, even if the final height does not ultimately reach the forecasted height.

In addition, we have shown that there is a probable effect on head circumference and a possible effect on motor and even cognitive function. A discrepancy between longitudinal and head circumference results may indicate that the reduced head circumference in DS is not only a result of growth retardation but is mainly due to limited brain growth.

Despite the great importance of the treatments’ effect on quality of life, no study has formally examined this aspect by accepted and valid questionnaires.

No significant adverse effect of GH treatment in DS patients was ever reported even in the long- term. Certainly, more research is needed to describe the effect of longer-term treatment, but the results of this review provide a strong basis for considering that the risks of GH therapy in children with DS are not significantly different from other children.

The literature on height outcome in DS pediatric patients, who were treated with recombinant human GH, is heterogeneous, with variable age at diagnosis, years of follow-up, and variable criteria and methods of height measures. Although the number of studies included in this mini meta-analysis is not large, it is important to note that to the best of our knowledge this review included all studies that examined the effect of GH treatment in children with DS, from whom a reliable and comparable measurement tool, like height SDS, could be derived. It is also noteworthy that even studies that could not be included in the analysis reached the same conclusions about the beneficial effect of GH therapy on the longitudinal growth of children with DS.

Recently, an original study assessed whether anthropometric measurements of children with DS correlate with their IQ. The results showed that full-scale, verbal, and nonverbal IQ correlated with height percentile in a multiple linear regression analysis. The results of this study suggest an association between growth and IQ in children with DS, and this finding may be valuable for this population in light of increasing access to GH therapy in various genetic syndromes associated with short stature (35). DS shares many common clinical features with other genetic syndromes such as Prader-Willi syndrome (PWS). Children with PWS are also treated by GH treatment starting from the first year of life. Early GH treatment has been shown to promote mental and motor development as well as adaptive functioning in the PWS population in addition to improving growth velocity and metabolism (36).

Heterogeneity indices were calculated and were found to be relatively high. It is reasonable to conclude that one study (23) is responsible for the most heterogeneity from looking at the forest plot graph and from other calculations. In this study, the initial height index was significantly lower compared to the other studies. However, the growth rate through the treatment period was very similar to the growth rate observed in the other studies, as can be seen from **Figure 2**.

GH treatment was found to be effective equally in DS children who were diagnosed with GH deficiency and in DS children who were not diagnosed with GH deficiency. This finding corresponds to the conclusion that despite the increased prevalence of GH deficiency in children with DS, the quantitative component of the growth hormone is only one of the possible damaged elements in the GHRH-GH-IGF1 axis in DS patients, and apart from that, an impairment was also detected in GH neurosecretory function and in the bio-activity of GH, both of which are likely to be missed if relying solely on growth hormone stimulation tests (4).

It should be noted that all three of the above components negatively affect the level of IGF1 in patients with DS. It was recently found that biomarkers of neurodegeneration are associated with IGF1 deficiency in DS and that short stature is associated with lower IGF1 and with higher biomarkers (37).

Exogenous growth hormone treatment is expected to provide a response to these three issues.

In contrast to more distal disturbances such as growth hormone receptor resistance and IGF-1 receptor deficiency, the administration of proper recombinant GH circumvents both the quantitative and qualitative disturbances in the production and secretion of GH, as well as any defects in the endogenous protein structure. It seems that a diagnosis of growth hormone deficiency in children with DS should not be a criterion for GH treatment.

### 4.2 Ethical considerations

The main ethical arguments that have been raised over the years concerning GH treatment of DS patients consider the safety and necessity of GH treatment, while also raising the issue of patient autonomy and agreement (**Table 1**).

In the “safety” category, the main health concern that has been raised is the fear of developing leukemia, importantly as DS patients have a predisposition to developing leukemia. The fear of developing leukemia arose at a time when there was a similar general concern about growth hormone treatment in the general population. Since then, large studies have already shown that this fear is unwarranted in the general population. Correspondingly, we have shown herein that there is no evidence of the development of leukemia in small studies, most of which have documented short-term follow-up but also in the minority that documented long-term follow-up.

In the “necessity” category, it has been argued that short stature does not constitute a significant limitation to DS patients necessitating the need for potentially harmful and expensive GH treatment because of their developmental intellectual disability. Also, growth hormone treatment is given every day by subcutaneous injection which involves discomfort for the patient. Identifying with the purpose of the treatment makes it easier for the patient to cope with the pain. In the absence of such identification, as in the case of a child with Down syndrome due to developmental delay, the child’s difficulty in dealing with the pain and the parent’s difficulty in providing the treatment may increase.

In the “agreement, justice, and autonomy” category, it has been argued that informed consent is difficult because many of these children are limited in their ability to understand issues involved with GH treatment. Further, there is concern of the psychologic effects of repeated GH injections to children who may not understand the purpose of the treatment and may view the treatment as an additional “punishment” to their condition.

The degree of developmental intellectual disability in DS patients is highly variable. Average IQ of standard DS is around 50 and that of mosaic DS is around 65, showing that most DS patients have a mild degree of developmental intellectual disability. A large survey in the US reported that 57 percent of adult DS people were working a paid job (38).

Gradual but dramatic changes over time in life expectancy, quality of life, social involvement, and functional level highlight some of the important values associated with GH treatment that should not be ignored. While GH treatment may not improve intellectual performance, by improving stature, it may affect a variety of factors which are beyond mere centimeters. It is well established that short stature impairs outlook for socialization, employment, general well-being, and happiness (39). Some parents of DS children have stressed the fact that restricting DS children of GH therapy risks layering handicaps on handicaps, ultimately depriving them of any chance of living a healthy and happy life as contributing members of society, since attaining a “normal” height and growth becomes just as important for children with DS as for any other child (16).

It seems ethically, morally, and legally right that children with DS receive the same treatment as any other child without bias or judgement, and pediatricians need to respect and consider the experiences of parents and their wishes regarding decisions about GH therapy. At the same time, GH responsiveness and DS diagnosis should not be an automatic indication for GH therapy, rather the decision should be made based on well-informed consultations with caregivers and their wishes after discussing benefits and potential risks of GH therapy and clarifying potential misconceptions.

### 4.3 Limitations and strengths

Inferences presented in this review are weakened by the risk of potential bias caused by the design of included studies. Any attempt at pooling the data should be done with caution to derive a clinically meaningful result. The relatively small total number of participants, especially the control groups, limits our ability to draw unambiguous conclusions.

On the other hand, the systematic nature of this review, which is not limited by language or a year limit, gives it its strength.

### 4.4 Conclusions

There is a significant short-term beneficial effect of GH therapy on longitudinal growth in children with DS. The presented findings may be valuable for improving access to GH therapy for pediatric DS patients. However, these findings should be confirmed by further research with a longitudinal sample of children with DS.

## Data availability statement

The raw data supporting the conclusions of this article will be made available by the authors, without undue reservation.

## Author contributions

DS, EH, and AH initiated the study and devised its main idea. AB and ST were invaluable in performing the meta-analysis. All authors contributed to the article and approved the submitted version.

## Supplementary material

SUPPLEMENTARY TABLE 1 GH Treatment mini meta-analysis - Main characteristics and findings of included studies.
