# Supplementary material for: GH treatment in pediatric Down syndrome: a systematic review and mini meta-analysis
Source: Front Endocrinol (Lausanne). 2023 Apr 21;14:1135768. doi: 10.3389/fendo.2023.1135768 (PMC10162572; doi:10.3389/fendo.2023.1135768)
Supplement: SUPPLEMENTARY TABLE 1 — GH Treatment mini meta-analysis - Main characteristics and findings of included studies. [file Table_1.docx]

| **Lead author & year**  **(Ref)** | **Participants no.,**  **GH Tx start age** | **Controls no.** | **Inclusion**  **criteria** | **Treatment type,**  **dosage, duration (months)** | **Height Outcome** | **Post Treatment effect on height & final height** | **Head circumference** | **Cognitive function, motor function,**  **additional effects** | **Adverse effects** | **IGF-1 levels** | **Bone age** |
| --- | --- | --- | --- | --- | --- | --- | --- | --- | --- | --- | --- |
| Berg J 1961 (10) | 3  Age 1-4 y | 3 | No intercurrent illness,  no specific motor defects | Pituitary extracts,  6 months | Non-significant | Non-significant | Non-significant difference in head circumference or in skull X-ray | No significant differences in terms of intelligence or behavior | Slight increase in mean neutrophil lobe count | Not assessed | Not assessed |
| Annerén G 1986 (11) | 5  Age 3-6 y | – | No clinically important congenital malformations.  None were malnourished.  All–height >3 SD below the mean according to the Swedish standard. | Human GH,  0.5 U/kg/wk (divided into 3 doses),  6 months | All the patients increased their growth velocity during treatment from roughly 50% to 200%. | After GH Tx termination, growth velocity essentially returned to the values before treatment. | Not assessed | Cognitive function- non-significant change | Slight increase in mean neutrophil lobe count. Increase in neutrophil count and a fall thereafter. | Were below normal range before treatment and increased to normal after 10 days of treatment on average and remained normal during treatment in all patients | Was delayed by 1.5 years on average. It was advanced by 0.5 year after treatment in 4 patients and by 1 year in 1 patient |
| Torrado C 1991(12) | 13  Age  1.5-4.9 y | – | No CHD. Pathologic low growth velocity for at least 1 year before treatment. Bone age >-1.5 SD for age. A formal diagnosis of GH deficiency was not required, but low peak GH response to 1 stimulation test or pathological ICGH was required. Stable family | rhGH,  0.1 mg/kg/wk (divided into 3 doses),  12 months | Significant increase in growth velocity and height SDS | Not assessed | Significant increase from -3.1 ± 1.3 SD to -2.6 ± 1.4 SD at 6 mo and to -2.3 ± 1.2 SD at 1 yr | not assessed | Two patients had elevated TSH levels. Mild transient elevations in serum prolactin  Weight SDS average before treatment was -1 and +0.6 after 1 year of treatment | Low normal before therapy and increased significantly during treatment | Was less than -2SD for age before treatment. The mean increment during the year of treatment corresponds to the increment in chronological age |
| Castells S  1996 (13) | 30  Age 1-11 y | 12 | No active CHD.  No evidence of psychological deprivation or abuse Prepubertal. At least one pathological GH stimulation test. | rhGH,  0.3 mg/kg/wk (divided into 3 doses),  6-88 months | Significant increase in growth maintained for as long as 6 years | Predicted adult height (PAH) was calculated during therapy and was 170.7 ± 10.8 cm in 19 males and in 167.1 ± 5.2 cm in 16 females. | Significant consistent increase during 6 years of treatment | Not assessed | No hyper-glycemia till 6 years after treatment. No case of pseudotumor cerebri or slipped capital femoral epiphysis or abnormal thyroid function. No changes in leukocyte count and morphology. | A dramatic significant increase from pre- to post treatment | Significant consistent increase during 4 years of treatment. |
| Wisniewski KE 1996 (14) | 40  Age 1-11y | 12 | No active CHD.  No evidence of psychological deprivation or abuse. Prepubertal. At least one pathological GH stimulation test | rhGH,  0.3 mg/kg/wk (divided into 3 doses),  6-88 months | Data presented in a former article | Data presented in a former article | 32 out of 40 were reversed from microcephaly to normocephaly only in children treated before the age of 5 years | Cognitive functioning level did not change significantly | Data presented in a former article | Data presented in a former article | Data presented in a former article |
| Annerén G 1993 (15) | 16  Age 6-9 mo | 40 | No congenital cardiac malformations, no thyroid dysfunction | rhGH, 0.033mg/kg/ day,  30 months | Significant increase in growth maintained after 30 months of treatment | Results have not yet been assessed | Slightly increased during the therapy | Results have not yet been assessed | In one girl, GH Tx was stopped after 1 year because of slightly increased serum level of liver enzymes. One girl developed celiac disease – unlikely due to GH Tx | Results have not yet been assessed | No acceleration of bone age development was observed the first-year treatment |
| Annerén G 1996 (16) | 15  Age 6-9 mo | 15 | No congenital cardiac malformations, no thyroid dysfunction | rhGH,  0.233 mg/kg/wk,  36 months | Significant increase in growth maintained after 36 months of treatment | Growth velocity 1 year after GH Tx was stopped- in some of the children – seemed to be reduced and in some, the increased continued | Very slight increased rate | Cognitive function Griffith’s test and Motor function- Motor-Perceptual test were performed at GH Tx start, 1 year after start, at end, and 1 year after end – preliminary results do not reveal any effect. | No late complications were reported | Increased rapidly when GH Tx was started, and the level of IgF1 was in the normal range during the whole GH Tx period | Average slightly decreased retardation after 3 years of treatment. in 7 out of 15 cases, bone age was slightly advanced |
| Annerén G 1999 (17) 2000 (18) | 15  Age 6-9 mo | 15 | No congenital cardiac malformations, no thyroid dysfunction | rhGH,  0.233 mg/kg/wk,  36 months | Data presented in a former article | 3 years after the end of treatment, there was a significant difference in height between the groups (p < 0.05) | There was a difference between the two groups at the start of the study, and this difference did not change during the period of treatment | No differences concerning mental development or gross motor development was observed between the control and treated groups when the GH Tx was ended. However, a somewhat better ﬁne motor performance was noted in the GH-treated children | No late complications were reported | Decreased during the year after the end of treatment, but to higher levels than the start –within the normal range | Somewhat retarded at the start of GH Tx, but was in the normal range after 3 years of treatment |
| Carlstedt K  1999 (19) | 10  Age 6-9 mo | 16 | No congenital cardiac malformations, no thyroid dysfunction | rhGH,  0.233 mg/kg/wk,  36 months | Data presented in a former article | Data presented in a former article | Data presented in a former article | 3 years after GH Tx termination, there were no differences concerning craniofacial development and dental age in DS children treated with GH or not | No late complications were reported | Data presented in a former article | Data presented in a former article |
| Myrelid Å 2010 (20) | 12  Age 6-9 mo | 10 | No congenital cardiac malformations, no thyroid dysfunction | rhGH,  0.233 mg/kg/wk,  36 months | Data presented in a former article | Approximately 15 years after GH Tx termination, ﬁnal height did not differ between the GH-treated subjects and the extended group of controls | Approximately 15 years after GH Tx termination, the treated patients had a significantly greater head circumference SDS than the extended group of controls | Approximately 15 years after GH Tx termination – no statistical preference to the GH Tx group in brief IQ scores but significant higher scores in all subtests of cognitive tests Leiter-R and WISC-III, and in all but one subtest of the motor BOT-2 test.  Adult weight did not differ between groups | Approximately 15 years after GH Tx termination, no late complications were reported. | Not assessed | Not assessed |
| Ragusa L 1996 (21) | 9  Age 1-9 y | – | GH deficiency | rhGH,  0.33 mg/kg/day,  6-36 mo | 7 out of 9 patients signific-antly improved their starting centile for height | Not assessed | Not assessed | Not assessed | No appreciable side effects.  No effect on HBA1C.  In some cases, subclinical hypothyroidism was treated with L thyroxine with prompt normalization of TSH values.  No appreciable side effects | Previously low levels reached normal range during GH Tx | Not assessed |
| Yasuhara A 2001 (22) | 1  Age 3y | – | Irrelevant  normal level of GH | rhGH,  0.023 mg/kg/day,  11 mo | Increased growth rate | Not observed | Not assessed | Dramatic improvement in gross motor function | No appreciable side effects | 17ng/ml | 3.0 by the TW-2 method standardized for Japanese children before treatment |
| Pallotti. S 2002 (23) | 10  Average  age 9.4y | – | No CHD.  Normal thyroid function.  Height SDS<-2.  GH deficiency.  Proportion between chronologic age to bone age – less than 1.5 | rhGH,  0.033 mg/kg/wk divided into 6 daily doses,  36 mo | Significant increase in growth rate in all patients | 10–15 years after the end of GH Tx, an improvement in final height of on average 5.16 cm in males and 7.35 cm in females compared to C. Cronk DS growth charts | Not assessed | Not assessed | 10–15 years after the end of GH Tx, no hematologic consequence, normal thyroid and liver function, normal HBA1C | Not assessed | Not assessed |
| Meguri K 2013 (24) | 20  Age 4.6±1.9y | – | GH deficiency  Seven children had history of congenital heart disease that could affect growth.  One had congenital hypothyroidism.  Two had hematologic diseases including acute myeloid leukemia, which had been cured before GH Tx initiation. | rhGH,    0.22 mg/kg/wk ,  Average duration 5.2 y. range: 1.0 to 10.4 y  In 3 patients, stopped after reaching final height.  6 patients were  still continuing their treatment in November 2012 | Height SDS increased significantly.  In patients receiving long-term GH Tx, the growth curves during therapy paralleled the standard curves overall, and growth persisted even in late puberty, while the DS standard curve flattens at that time | Not assessed | Not assessed | The effects of GH on body composition are hard to distinguish from natural processes of growth, and clear conclusions cannot be drawn. Mean BMI remained unchanged from baseline to 3 yrs. after initiation of GH Tx | Precocious puberty was observed in 1 boy 3.4 yr after GH Tx initiation.  Otitis media was reported in 4 children.  Growing pain was reported in one patient. Neither of these adverse events were related to GH Tx.  TSH was elevated in 1 boy who received oral levothyroxine.  No clinically significant changes in CHD diseases | Not assessed | No excessive progression in bone age was noted when the values were compared with chronological  age |
